# Anxiolytic and Antidepressant-Like Effects of the Aqueous Extract of *Alafia multiflora* Stem Barks in Rodents

**DOI:** 10.1155/2012/912041

**Published:** 2012-10-22

**Authors:** Harquin Simplice Foyet, David Emery Tsala, Armand Abdou Bouba, Lucian Hritcu

**Affiliations:** ^1^Department of Agriculture, Livestock and By-Products, The Higher Institute of the Sahel, University of Maroua, P.O. Box 46, Maroua, Cameroon; ^2^Department of Life and Earth Sciences, Higher Teachers' Training College, University of Maroua, P.O. Box 55, Maroua, Cameroon; ^3^Department of Biology, Alexandru Ioan Cuza University, Bou/evard Carol I 11, 700506 Iasi, Romania

## Abstract

The present study examined the anxiolytic and antidepressant effects of the aqueous extract of *Alafia multiflora Stapf* (AM) stem barks (150 and 300 mg/kg, 7 days administration) on rats and mice, using experimental paradigms of anxiety and depression. In the open field, the aqueous extract increased significantly the number of center square crossed and the time spent at the center of the field as well as the rearing time, while the grooming time was reduced significantly. In the elevated plus maze, the aqueous extract increased the time spent and the number of entries in the open arms. All these effects were also completely reversed by flumazenil, an antagonist of benzodiazepine receptors and pindolol a **β**-adrenoceptors blocker/5-HT 1A/1B receptor antagonist. The time spent in the light compartment, the latency time, and the number of the light-dark transitions increased significantly in the light/dark exploration test after the treatment with AM. The extract was able to reduce significantly the immobility time and increase swimming as well as climbing duration. Taken together, the present work evidenced anxiolytic effects of the aqueous extract of AM that might involve an action on benzodiazepine-type receptors and an antidepressant effect where noradrenergic mechanisms will probably play a role.

## 1. Introduction

Anxiety and depressive disorders are frequent psychiatric conditions identified as the most common stress-related mood disorders causing disability and premature death. More than 20% of the adult population suffer from these conditions at some time during their life [1]. The World Health Organization envisaged that depression will become the second leading cause of premature death or disability worldwide by the year 2020 [2]. The complexity of daily life in modern society leads to various degrees of anxiety and depression. Mood, depression, and anxiety disorders have been found to be associated with chronic pain among medical patients in both developed and developing countries [3]. For many years, they were considered as two different mental diseases, with the benzodiazepines used as the drugs of choice for acute anxiety states and the amine uptake inhibitors and monoamine oxidase inhibitors to treat depression. However, in the clinical practices of the treatment of anxiety disorders, benzodiazepines are now slowly replaced by antidepressants, which are not only efficacious in depression but also in the acute and chronic treatment anxiety disorders [1].

The GABAergic system and the serotoninergic neurotransmission are involved in anxiety. In addition, selective serotonin reuptake inhibitors (SSRIs) are effective in anxiety disorders and are known to have strong antidepressant effects [4, 5]. Depression is related to monoamines in the brain, especially to 5-HT and noradrenaline. Although benzodiazepines have well-known benefits, their side effects are prominent, including muscle relaxation, sedation, physical dependence, memory disturbance, and interaction with other drugs [6]. In these conditions, the efficacy of such drugs is very limited so the need for newer, better-tolerated, and more efficacious treatments remains high. 

Herbal therapies could be considered as alternative or complementary medicines. In the search for new molecules useful for the treatment of neurological disorders, worldwide medicinal plant research has continued to progress, demonstrating the pharmacological effectiveness of different plant species in a variety of animal's models [7]. This is reflected in the large number of herbal medicines whose psychotherapeutic potential has been assessed in a variety of animal models. These studies have provided useful information for the development of new pharmacotherapies from medicinal plants and for new isolated active phytoconstituents. 

*Alafia multiflora* (Apocynaceae) is a medicinal plant widely distributed in the tropical region of Africa, traditionally used for ulcerous wounds and occasionally used for abdominal pain. Previously, we showed the protective effect of the methanol/methylene chloride extract of this plant on carbon tetrachloride- (CCl_4_-) induced oxidative stress in rats. Furthermore, antibacterial and antiradical activities of different extracts of this plant have been demonstrated along with a high LD_50_ (>5 g/kg) in rats. Recently both biochemical and histopathological studies in rats demonstrated that the methanolic extract of *A. multiflora* at doses of 125 and 250 mg/kg has hepatoprotective activity due to its antioxidant potential [8]. Phytochemical screening of the stem bark showed the presence of phenols, tannins, flavonoids, anthraquinones, and alkaloids [9]. 

A wide range of plant-derived flavonoids, terpenes, can cross the blood-brain barrier and are able to influence brain function [10] such as the modulation of the function of ionotropic GABA receptors. Due to the presence of flavonoids in the extract of *A. multiflora* and its higher antioxidant activities, it is presumed that this plant might have benefic pharmacological effects at the level of the central nervous system. 

Therefore, the objective of the present work was to analyse the possible anxiolytic and antidepressant-like effects of the aqueous extract of *A. multiflora* stem bark in rats and mice using the open field, elevated plus-maze and light-dark box tests as animal models of anxiety, and forced swimming test as an animal model of depression, respectively. 

## 2. Materials and Methods

### 2.1. Plant Material and Extraction

Plant material (stem bark) was collected in the centre region of Cameroon in May and authenticated at the National Herbarium-Yaoundé, where the voucher specimen was conserved under the reference number 43196/HNC. Aqueous extract was prepared as follows: after drying fresh stem bark and powdering it, 900 g of the powder were dissolved in boiled distilled water (1 litre) for 24 hours. This was followed by filtration and elimination of the solvent under air-dried oven at 50°C. The given powder yielded 3.24% of a dark brown extract.

### 2.2. Experimental Animals

Wistar albino rats (weighing 160–180 g) and Swiss albino mice (weighing 20–25 g) of both sexes were obtained from the veterinary national laboratory (LANAVET) of Garoua, Cameroun. The animals were housed in polyacrylic cages (6 animals/cage) and maintained in a temperature and light-controlled room (25 ± 2°C, a 12 h cycle). The animals were acclimatized to laboratory condition for 10 days before the start of experiment. Prior to and after treatment, the animals were fasted for 12 and 7 h, respectively. However, all animals were allowed to drink water *ad libitum*. The authorization for the use of laboratory animals in this study was obtained from the Cameroun National Ethical Committee (Registration number: FWA-IRB00001954).

### 2.3. Chemicals

Diazepam hydrochloride, pindolol, flumazenil, and fluoxetine were purchased from Sigma-Aldrich Co., USA, and used as a reference drugs. All drugs and extracts were freshly prepared in saline on the day of the experiments and administered intraperitoneally (i.p.). Control animals received 10 mL/kg body of the vehicle in the same route of administration.

## 3. Behavioral Evaluation

### 3.1. Open Field Activity Test (OFT)

The open field apparatus was constructed of white polywood and measured 72 × 72 cm with 36 cm walls. Red lines were drawn on the floor with a marker and were clearly visible through the clear Plexiglas floor. Mice were injected (i.p) with the aqueous extract of *A. multiflora* stem bark once per day for 7 days. The test was performed 30 min after the last administration of the aqueous extract of *A. multiflora* stem bark (150 and 300 mg/kg, i.p.) or saline (10 mL/kg). The standard drug diazepam (1 mg/kg, i.p.) was given once 30 min before the test. The mice were placed in the open field box for 6 min, and their behaviors were recorded. The behaviors scored included time spent at the center square, number of the lines crossed in the floor of the maze, rearing frequency (number of times the animal stood on its hind legs), and grooming (duration of time the animal spent licking or scratching itself while stationary) [11]. 

### 3.2. Elevated Plus-Maze Test (EPM)

Behavior in the elevated plus maze (EPM) is used to assess exploration, anxiety, and motor behavior. The possible anxiolytic effects of the aqueous extract of *A. multiflora* stem bark were assessed, basically using the same method described by Foyet et al. [12]. The EPM consists of four arms, 49 cm long and 10 cm wide, arranged in such a way that the two arms of each type were opposite to each other. The maze was elevated 50 cm above the floor. Two arms were enclosed by walls 30 cm high and the other two arms were exposed. Rats were injected i.p. with the aqueous extract of *A. multiflora* stem bark (150 and 300 mg/kg, i.p) or saline (10 mL/kg, i.p) once per day for 7 days. The positive control diazepam (1 mg/kg, i.p) was given once 30 min before the test. Thirty minutes after the i.p. injection of the last dose of extract or saline, each animal was placed at the center of the maze facing one of the enclosed arms. During a 5 min test period, the number of open and enclosed arms entries, as well as the time spent in open and enclosed arms, was recorded as previously described [12, 13]. Entry into an arm was defined as the point when the animal places all four paws into the arm. After the test, the maze was carefully cleaned with 10% ethanol solution and allowed to dry before the next animal.

In another set of experiments, rats were subjected to the coadministration of the aqueous extract of *A. multiflora* leaves and pindolol or flumazenil. Thirty minutes before the oral administration, the plant extract (150 and 300 mg/kg), pindolol (10 mg/kg), flumazenil (10 mg/kg), or vehicle (10 mL/kg) were administered intraperitoneally. Two other groups of rats were treated with pindolol or flumazenil during the same period [14].

### 3.3. Light-Dark Transition Test (LDB)

The LDB test was performed according to the method of Gong et al. [15], with minor modifications. Our light/dark box (45 × 27 × 27 cm) was made of polywood and consisted of two chambers connected by an opening (7.5 × 7.5 cm) located at the floor level in the center of the dividing wall. The floor was divided into 9 × 9 cm squares and was covered with Plexiglas. The small chamber (18 × 27 cm) was painted black and the larger chamber (27 × 27 cm) was painted white. Bright illumination was provided by a 60 watt table lamp located 40 cm above the center of the white chamber. Mice were injected (i.p) with the aqueous extract of *A. multiflora* stem bark once per day for 7 days. The test was performed 1 h after the last extract administration. Standard drug diazepam (i.p.) was given once 30 min before the test. During the test, the mice were placed at the center of the light compartment with their back to the dark compartment, and then transition behavior over 10 min was observed, including the latency time (latency before entering the dark compartment), the transition number (the number of dark compartment to light compartment transitions), and the total time spent visiting the light compartment [16, 17]. After 5 min, mice were removed from the box by the base of their tails and returned to their home cage. The maze was then cleaned with a solution of 10% ethanol and permitted to dry between tests.

### 3.4. Forced Swimming Test (FST)

The FST is the most widely used pharmacological model for assessing antidepressant activity [18]. The development of immobility when the rodents are placed in an inescapable cylinder of water reflects the cessation of persistent escape-directed behavior [19]. The possible antidepressant effects of the aqueous extract of *A. multiflora* stem bark were assessed, basically using the same method described by Foyet et al. [12] with minor modifications. Rats were administered with aqueous extract of *A. multiflora* stem bark (150 and 300 mg/kg, i.p) or saline (10 mL/kg, i.p) once per day for 7 days. The standard drug fluoxetine (10 mg/kg, i.p) was given once 30 min before the test. On the first day of the experiments (pretest session), rats were individually placed into transparent Plexiglas cylinder (50 cm high and 20 cm wide) filled to a 30 cm depth with water at 26 ± 1°C. The animals were left to swim for 15 min before being removed, dried, and returned to their cages.

 The procedure was repeated 24 h later, in a 6 min swim session (test session) 30 min after the last dose of the extract of *A. multiflora* stem bark, fluoxetine, or saline. During the test session, the following behavioral responses were recorded: immobility time (time spent floating with the minimal movements to keep the head above the water), swimming time (time spent with active swimming movements), and climbing time (time spent with upward movements of the forepaws directed to the cylinder wall). Increases in active responses, such as climbing or swimming and reduction in immobility, were reconsidered as behavioral profiles consistent with an antidepressant-like action [18].

### 3.5. Statistical Analysis

Data were presented as mean ± SEM values. One-way ANOVA with Dunnett's posttest was performed using Graph Pad Prism version 5.00 for Windows, Graph Pad Software, San Diego, CA, USA, www.graphpad.com. A probability level of 0.05 or less was accepted as significant.

## 4. Results

### 4.1. Effects of the Extract in the OFT

The open field test was performed for 30 min after the administration of the last dose of the extract or 30 min after single dose of diazepam. The extract did not significantly increase the number of lines crossed by the mice at any dose tested, but did significantly increase rearing time at the 300 mg/kg dose. In contrast, diazepam significantly decreased both the number of lines crossed and rearing time. The grooming time was significantly reduced by the extract and the standard drug, compared with the control group. Diazepam significantly increased the time mice spent at the center of the field. Moreover, this parameter was also significantly increased by the chronic administration of the extract (Table 1).

### 4.2. Effects of the Extract in the EPM

**Figure 1:**
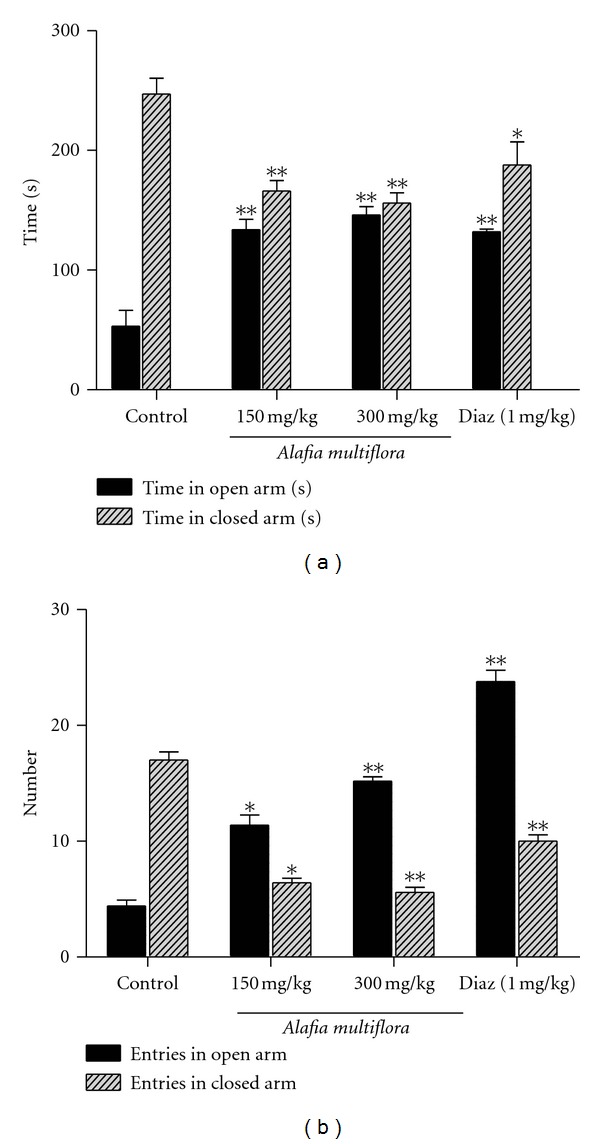
Effect of the aqueous extract of *A. multiflora* stem bark on the time spent in open and closed arms (a) and the numbers of entries in open and closed arms (b) in the elevated maze test. Experiments were performed 30 min after the last chronic administration of the aqueous extract *of A. multiflora* or 30 min after the single dose of diazepam (Diaz) administration. Data are expressed as mean ± SEM of 6 animals. *P < 0.05, **P < 0.01, compared to the vehicle-treated control group.

The extract at doses of 150 and 300 mg/kg i.p. produced anxiolytic-like effects as determined by the increase of the time spent in the open arms compared to control animals (*P* < 0.001, Figure 1(a)). Consequently, the plant extract significantly (*P* < 0.05, Figure 1(b)) increased the number of entries in the open arms of the plus maze. Conversely, the time spent and the numbers of entries in the enclosed arms were reduced by the extract treatment compared to control animals (Figures 1(a) and 1(b)).

### 4.3. Effect of Pindolol and Flumazenil Antagonism on the Anxiolytic Effect of the Extract

**Figure 2:**
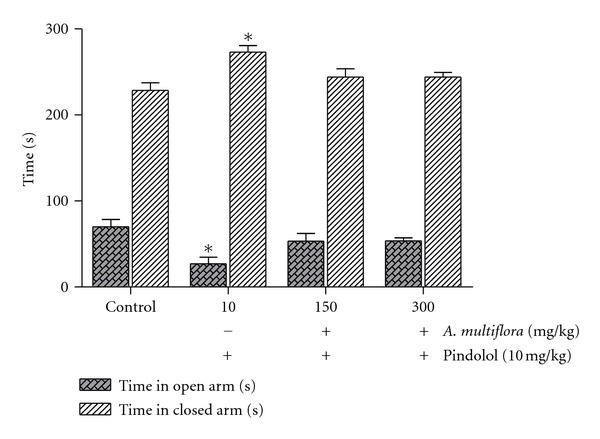
Antagonistic effect of pindolol on the anxiolytic-like effect of the aqueous extract of *A. multiflora* stem bark. Pindolol (10 mg/kg) was given 30 min before the extract and during the same period. Data are expressed as mean ± SEM of the time spent in the closed and open arms in rats given 5 min test 30 min after the last administration of the extract. *P < 0.05, compared to the vehicle-treated control group, *n* = 6 rats per group.

**Figure 3:**
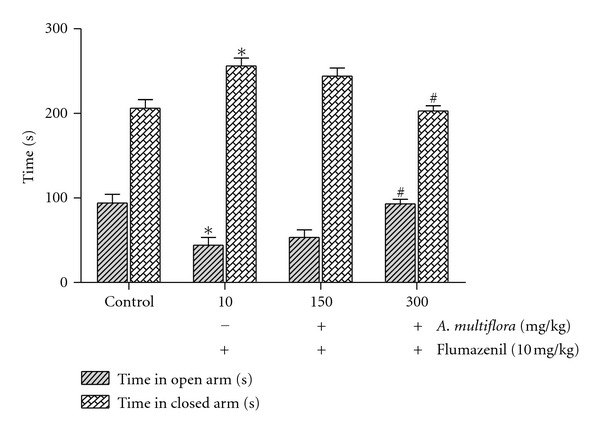
Antagonistic effect of flumazenil on the anxiolytic-like effect of the aqueous extract of *A. multiflora* stem bark in rats. Flumazenil (10 mg/kg) or vehicle was administered intraperitoneally. The data is expressed as the mean ± SEM of the percentage of the time spent and the number of entries into the open arms of the elevated plus maze over 5 min test period. *P < 0.05 compared to the vehicle-treated control group; ^#^ *P* < 0.05 versus the flumazenil treated group, *n* = 6 rats per group.

As shown in Figures 2 and 3, the behavioral effects of the aqueous extract of *A. multiflora* stem bark were completely antagonized by pindolol (10 mg/kg) and flumazenil (3 mg/kg).

### 4.4. Effects of the Extract in the LDB

The extract at 150 mg/kg significantly increased the latency time, the number of the light-dark transitions, and the time spent in the light compartment. Diazepam (1 mg/kg, i.p) significantly increased the number of transitions and the time spent in the light compartment while the latency time significantly decreased (Table 2).

### 4.5. Effects of the Extract in the FST

**Figure 4:**
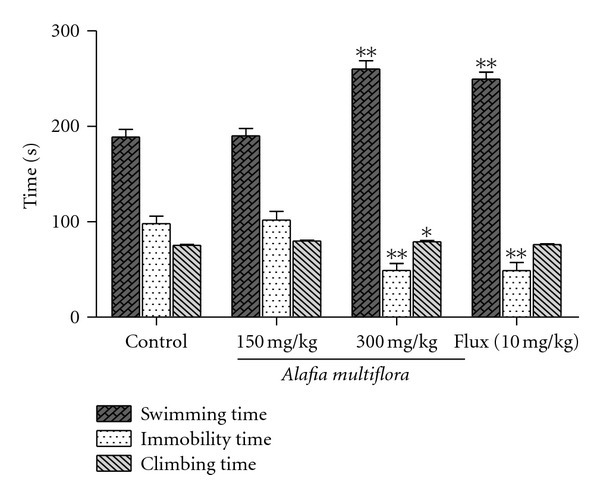
Effects of the aqueous extract of *A. multiflora* stem bark or fluoxetine on the forced swimming test in rats. Animals were treated with the extract (150 mg/kg or 300 mg/kg, i.p.) or distilled water once a day for 7 days. In the positive control, fluoxetine (Flux) was given only once (10 mg/kg, i.p.) 30 min prior to the test. Each column represents the mean ± SEM of 6 animals. Data analysis was performed using Dunnett's *t*-test, *P < 0.05; **P < 0.01 significantly different from saline-treated animals.

The impact of the extract on immobility time in the forced swimming test in rats is shown in Figure 4. After 7 days i.p. administration of this extract, the immobility time was significantly reduced in rats that received a daily dose of 300 mg/kg but not 150 mg/kg. This activity was similar to that of fluoxetine at 10 mg/kg. Conversely, the swimming and the climbing time (Figure 4) were significantly (*P* < 0.01 and *P* < 0.05, resp.) increased by the aqueous extract of *A. multiflora* stem bark. Fluoxetine had no significant effect on climbing time, but did increase swimming time.

## 5. Discussion 

In the present study, the anxiolytic and antidepressant-like effects of the chronic administration of the aqueous extract of *Alafia multiflora* stem bark were studied in different animal models of anxiety and depression. The *Alafia multiflora* stem bark extract was first studied using the open field which gives a better indication of the animal's emotional state. The administration of the plant extract and diazepam produced a significant reduction of the grooming time and an increase in the time spent at the centre of the field. Grooming behavior following exposure to stress and the increase of the time at the center of the field clearly indicate that the plant extract has anxiolytic activity. The *Alafia multiflora* spontaneous activities were studied by rearing and the number of line crossed. The rearing (vertical movement) is an index of the locomotor activity [20] while the increased number of line crossed (horizontal movement) is an indication of the central nervous system stimulant properties. The chronic administration of the aqueous extract of the *Alafia multiflora* stem bark significantly increased the rearing time and the number of crossings. These results taking together indicate that, in contrast to diazepam, the aqueous extract of *Alafia multiflora* showed anxiolytic-like effects without affecting locomotor activity or without producing central nervous depression. However, the significant decrease in the number of line crossed by animals treated with diazepam suggests a sedative effect of this drug at the dose used.

One of the most widely used animal models for screening putative anxiolytics is the elevated plus maze, in which rodents display an avoidance of exposed open areas of the maze, which are presumed to be the most aversive, and a preference for sections enclosed by protective walls [21]. The anxiolytic effectiveness of a drug can be demonstrated by a statistically significant increase in rodent activity in the open arms. In the elevated plus maze test, the aqueous extract of *Alafia multiflora *increased but not in a dose-dependant manner, the time spent, and the numbers of entries in the open arms (*P* < 0.001). This anxiolytic effect of *Alafia multiflora* is similar to the one observed with diazepam, a typical benzodiazepine drug which induced significant increase in open arm time and in number of entries into the open arm. The effect of anxiolytic agents is to enhance the response to GABA, by facilitating the opening the GABAA-activated chloride channels. It can therefore be hypothesized that *Alafia multiflora* may be acting like a benzodiazepine-like substance. Supporting this view, the treatment with flumazenil, a specific antagonist of the benzodiazepine site in the GABA-BDZ receptor complex, at the dose of 10 mg/kg, was able to block significantly the anxiolytic effect induced by the *Alafia multiflora* extract and the diazepam. The benzodiazepines receptor agonists are commonly prescribed for the treatment of anxiety, sleep, and seizure disorders for over 40 years. Based on this antagonism study, it could be concluded that the aqueous extract of *Alafia multiflora* may probably work via the activation of the benzodiazepine site of the GABA receptors in the central nervous system. Similar results were obtained by Yu et al. [22], testing the anxiolytic-like effects of *Cinnamomum cassia* in mice. In the other hand, the anxiolytic effect of the aqueous extract of *Alafia multiflora* stem bark was significantly reversed by pindolol. Pindolol blocks 5-HT_1A_ autoreceptors, thus inhibiting the negative feedback produced by the rapid increase of 5-HT resulting from the blockade of 5-HT transporters [23]. Therefore, we think that the anxiolytic-like effect of *Alafia multiflora* is also mediated by the 5-HT_1A_ receptor. However, it is known that one of the pharmacological actions of pindolol include the blockade of the *β*-adrenergic receptor activity. Thus, a definitive conclusion that the anxiolytic activity of the plant extract is mediated through 5-HT_1A_, in addition to GABA, receptors cannot be made at this time. 

The present study also characterized the effects of the aqueous extract of *A. multiflora *stem bark, on rat's performance in forced swimming test (FST) following short-term (7 days) treatment. In this experiment, the immobility displayed by rodents when subjected to unavoidable stress such as forced swimming is thought to reflect a state of despair or lowered mood, which is thought to reflect depressive disorders in humans [12]. The immobility time has been shown to be reduced by treatment with antidepressant drugs [24]. Because the pharmacotherapy of depression typically requires chronic drug treatment to obtain a full response in terms of antidepressant effect, it is critical to perform repeated treatments in the FST rat model [25]. 

Reduction of immobility was comparable to that observed after i.p. administration of fluoxetine (10 mg/kg), the reference antidepressant drug. The decrease in immobility induced by fluoxetine is generally accompanied by an increase in swimming, whereas climbing duration was not affected by this drug [26]. It is widely known that swimming is sensitive to serotoninergic compounds, such as the selective serotonin reuptake inhibitor fluoxetine while the climbing behavior is sensitive to tricyclic antidepressants and drugs with selective effects on catecholamine transmission [27]. Taken this into account, the results obtained in this study strongly suggested the implication of the serotoninergic and catecholaminergic pathway in the antidepressant effect of the aqueous extract of *A. multiflora.*

The biological effects of the aqueous extract of *A. multiflora* observed in this study might be attributed to phytoconstituents in the plant. Our preliminary phytochemical screening revealed the presence of flavonoids and polyphenols in the extract of this plant [9]. It has been reported that some flavonoids bind with high affinity to the benzodiazepine site of the GABA receptor [28, 29]. Their general bioavailability and particularly their presence in the brain *in vivo* appear to play an important role in the expression of their effects on the CNS [12]. It is possible that the presence in the aqueous extract of the stem bark of *A. multiflora* of flavonoids could account for its effects on the CNS [30, 31]. 

The use of two types of animal species was simply due to lack of animals at the time in our institution. The behavioral field has adapted tests developed in the rat to the mouse with success and some tests like OFT and LDB have been easily retooled and validated for the mouse [32, 33]. Thus, we believe that the use of mice instead of rats does not fundamentally alter the trends of the results obtained in this study. 

In conclusion, our results provide evidence that the aqueous extract of the stem bark of *Alafia multiflora* possesses anxiolytic and antidepressant properties in rodents with no significant decrease in locomotor activity. Further neurochemical studies are necessary to elucidate the influence of this extract on the monoamine systems (5-HT, NA, and DA), which are critically involved in the development of clinical depression.

## Figures and Tables

**Table 1:** Effects of the aqueous extract of *A. multiflora* stem bark and diazepam in the open field test in mice.

Groups	Dose (mg/kg)	Number of line traversed	Rearing time (s)	Grooming time (s)	Time spent at the center (s)
Control	—	35 ± 5.2	23.54 ± 5.14	87.54 ± 12.62	5.70 ± 1.36
AM	150	49.12 ± 2.24	29.25 ± 3.46	57.32 ± 13.17	41.22 ± 5.61*
AM	300	48.21 ± 3.56	35.42 ± 2.20*	34.20 ± 6.16**	56.50 ± 4.04*
Diazepam	1	5.40 ± 4.32***	9.10 ± 3.10***	10.70 ± 8.56***	123.00 ± 18.120**

Mice activity was measured 30 min after the last chronic administration of the aqueous extract *of A. multiflora* or 30 min after the single dose of diazepam administration. Data are expressed as mean ± SEM of 6 animals. *P < 0.05, **P < 0.01, and **P < 0.001, compared to the vehicle-treated control group.

**Table 2:** Effects of repeated administration of the aqueous extract of *A. multiflora* (i.p.) on the light-dark transition test with mice.

Groups	Dose (mg/kg)	Latency time (s)	Transition number	Time spent in light compartment (s)
Control	—	116.2 ± 12.16	3.4 ± 0.73	38.60 ± 2.26
AM	150	196.60 ± 8.90***	7.2 ± 0.86**	53.80 ± 3.13*
AM	300	181.20 ± 11.20***	8.8 ± 0.86***	83.60 ± 4.06***
Diazepam	1	57.40 ± 3.73***	15.40 ± 1.20***	105.00 ± 3.33***

Aqueous extract of *A. multiflora* stem bark was given (i.p) once per day for 7 days. Diazepam was given (i.p.) only once 30 min prior to the test. Data are expressed as means ± SEM. *n* = 6 animals per group. *P < 0.05; **P < 0.01; ***P < 0.001 versus control.
